# Oscillation control of carbon nanotube mechanical resonator by electrostatic interaction induced retardation

**DOI:** 10.1038/srep22600

**Published:** 2016-03-03

**Authors:** Masaaki Yasuda, Kuniharu Takei, Takayuki Arie, Seiji Akita

**Affiliations:** 1 Department of Physics and Electronics, Osaka Prefecture University, 1-1 Gakuen-cho, Naka-ku, Sakai 599-8531, Japan

## Abstract

Despite the superb intrinsic properties of carbon nanotube mechanical resonators, the quality factors at room temperature are 1,000 or less, even in vacuum, which is much lower than that of mechanical resonators fabricated using a top-down approach. This study demonstrates the improvement of the quality factor and the control of nonlinearity of the mechanical resonance of the cantilevered nanotube by electrostatic interaction. The apparent quality factor of the nanotube supported by insulator is improved drastically from approximately 630 to 3200 at room temperature. Results show that retardation of the electrostatic force induced by the contact resistance between the nanotube and the insulator support improves the quality factor. Finite element method calculation reveals that the nonuniform pileup charge on the insulator support strongly influences the nonlinearity of the resonance.

Nanoscale mechanical resonators^1^,^2^,^3^,^4^,^5^ are widely used for highly sensitive force sensing, where the light weight and high resonance frequency are appropriate for higher sensitivity. Carbon nanotube (CNT) resonators^6^,^7^,^8^,^9^,^10^,^11^,^12^,^13^,^14^,^15^,^16^,^17^,^18^,^19^,^20^ are strong candidates for these applications because of their minute mass and high stiffness. The mass resolutions of CNT mechanical resonators have been achieved in the order of ~zg^14^,^17^ or less.^9^ The quality factor (Q-factor) is known as another important figure of merit for mechanical resonators. For these mass sensing applications of the mechanical resonators, a higher Q-factor is desired for higher resolution. Many efforts have been undertaken to improve the Q-factor of CNT mechanical resonators^21^,^22^. Recently, a very high Q-factor of approximately 5 × 10^6^ at quite low oscillation amplitude of CNT mechanical resonators^22^ has been achieved at the low temperature of 30 mK in high vacuum using advanced techniques of ultra-low-noise measurement. Despite the superb intrinsic properties of CNT mechanical resonators, the Q-factors of the CNT mechanical resonators at room temperature are 1,000 or less, even in vacuum, which is much lower than that of mechanical resonators fabricated using a top-down approach such as diamond^23^ or Si_3_N_4_^24^. Consequently, the extrinsic Q-factor control is indispensable to enhance the sensitivity of CNT mechanical resonators. Active Q control method^25^,^26^ is widely used to enhance the Q-factor of micro-fabricated cantilever for atomic force microscopy. Passive Q control^27^,^28^ is also widely investigated to ascertain whether to enhance or depress the mass-spring- damper system vibration. In this case, retardation effects on the velocity field of the oscillating system are induced by the external potential field, which results in modulation of the extrinsic Q-factors. Furthermore, nonlinear effects on mechanical resonators, which are commonly described by the Duffing equation at large oscillation amplitude, are important for the improvement of sensitivity or other applications such as bi-stable operation of nano-mechanical memory^10^,^29^,^30^,^31^. This study demonstrates Q-factor control using electrostatic attraction with retardation induced by the pileup charge on an insulator substrate near the CNT mechanical resonator inside a scanning electron microscope (SEM). In addition, the nonlinear effect on the CNT mechanical resonator is investigated based on the nonuniform electric field because of the pileup charge.

Figure 1a presents a schematic illustration of the cantilevered CNT mechanical resonator, where a CNT cantilever is supported by a SU-8 photoresist. First, CNTs were sandwiched by SU-8 layers on a Si substrate, where CNTs with electrical resistivities ranging from 2 to 20 μΩ·m examined in this study, synthesized using chemical vapor deposition (CVD), are highly graphitized and have a less amorphous carbon layer^32^ because of their post-annealing treatment at temperatures higher than 1500 °C. Subsequently, an optical lithography process using a focused laser was performed to fabricate the CNT cantilever as shown schematically in Fig. 1a. Therefore, one can fabricate CNT mechanical resonators supported by the insulator substrate through the simple photolithographic process (see supporting information for details of fabrication procedure). As a result, the electric potential of the CNT cantilever would be in almost floating because of the insulator support of SU-8.

The fabricated CNT cantilever was set on a 0.1-mm-thick piezoelectric actuator (PbZr_x_Ti_1−x_O_3_ ceramic) for oscillation by application of AC voltage with amplitude *V*_amp_ = 1–3 V in the SEM (~10^−4^ Pa). The frequency response of the CNT mechanical resonator was measured from a series of SEM images during the sweep of the drive frequency. Figure 1b,c portray SEM images for off-resonance and resonance of the CNT mechanical resonator examined in this study. Because the pileup charge on the insulator is strongly dependent on the material properties and surface condition of the insulator, it is hard to estimate the accurate pileup charge on the insulator, quantitatively. For this study, we use the electron dose estimated from the electron beam current on the substrate and the irradiation time as a guide for the pileup charge on the insulator. It is noteworthy that a certain electric charge is accumulated on the SU-8, CNT and native oxide on the Si substrate as a result of SEM observations, even immediately after the measurement set-up process. Consequently, it is impossible to obtain the frequency response for a no-pileup charge condition in SEM measurements.

Regarding control experiments without or less charge accumulation on the SU-8, optical measurements were performed in vacuum (~10^−4^ Pa) at room temperature, where the frequency response of scattered light from the vibrating CNT cantilever irradiated by the focused laser beam were measured through an optical aperture^13^,^17^,^33^. Figure 1d presents the frequency responses of the CNT cantilever measured using SEM and optical methods. The Q-factor without the e-beam charge accumulation obtained from the optical method is ~630, which is comparable to the reported values for CNTs supported on the conductive substrate measured from the SEM image^18^,^19^,^34^, where the nonlinear response was hardly observed. The Young’s modulus used for this experiment estimated from the resonance frequency is 0.2 TPa, where the diameter and length are, respectively, ~100 nm and 9.3 μm.

In contrast to the optical measurements, the Q-factor of SU-8-supported CNT obtained from the SEM measurements with the electron dose of 7 nC is ~3200, which is much larger than that measured by the optical measurement (see Fig. S2 in “Supporting information” for reproducibility). The tensile stress on the string type resonator is well known to improve the Q-factor. In this case, the resonance frequency *f*_0_ also increases concomitantly with increasing tensile stress. However, in our case, the resonance frequency shifts downward from 1687 kHz to 1591 kHz even with improvement of the Q-factor. Note that the Q-factor enhancement observed here has never observed in the case of a “conductive” support connected to a ground plane corresponding to no pileup charge and the resonance property was almost identical measured by the optical method (see Fig. S3 in “Supporting information” for one of examples). Additionally, the overdose condition of the electron beam causes permanent sticking of the cantilevered CNT to the SU-8 because of the excess electrostatic attraction, which indicates that the electrostatic attraction induced by the pileup charge certainly acts on the CNT cantilever (see Fig. S4 in “Supporting information” for more detail of the electrostatic attraction induced by the e-beam irradiation). A possible mechanism of the downshift of the resonance frequency might be nonuniform electric field induced by the pileup charge, which is similar to the electro-softening effects discussed commonly in micro-electro-mechanical system (MEMS) or SPM probe^25^,^26^. The improvement of Q-factor is also expected to be induced by the electrostatic force produced by the charge accumulation on the insulator support. However, the static electric field (no time dependence) even with nonuniform distribution cannot explain the Q-factor improvement observed here. Note that the kink structure observed in SEM images shown in Fig. 1b induces very few nonlinear or Q- enhancement effects under small vibration amplitude regime in our experimental condition, because the CNT with kink structure can be treated as a single spring consisting of a series connection of tiny springs with different spring constants (see “Supporting information” for more detail).

To elucidate the improvement of the Q-factor under the presence of the pileup charge *Q*_s_ on the insulator, the simplified equivalent circuit of this experimental setup shown in Fig. 1e is considered, where capacitance composed of the SU-8, *C*_SU-8_, is much greater than that of the oscillating CNT resonator, *C*_CNT_, which results in the almost constant voltage at the SU-8 surface, *V*_s_ = *Q*_s_/*C*_SU-8_, even with CNT cantilever oscillation. As described above, the CNT cantilever support was slightly embedded in the SU-8 layer. Therefore, one can assume that the CNT cantilever is connected to the constant voltage source, *V*_s_, through a certain contact resistance, *R*_C_. Capacitance *C*_CNT_ is modulated during the oscillation because the distance between the CNT cantilever and the substrate is changed depending on the oscillating position of the cantilever. The induced charge at *C*_CNT_ responds with certain delay time determined from *R*_C_ and *C*_CNT_, which induces the retardation effect on the CNT cantilever supported by SU-8. Note that the CNT cantilever would also be charged by the electron beam irradiation, we should consider the surface charge difference between the CNT cantilever and surrounding insulator substrate to obtain the electrostatic force acting on the CNT cantilever. Thus, we assume that the no pile-up on the CNT cantilever induced by the electron beam irradiation for simplified analysis.

We further assume that capacitance *C*_CNT_ of the harmonically oscillating CNT cantilever with frequency ω at a certain time *t* can be expressed as *C*_0_ + *C*_1_*e*^*iωt*^ under the condition of small oscillation amplitude, where *C*_0_ and *C*_1_ respectively stand for the DC and AC components of *C*_CNT_. Under these circumstances, the induced electrical potential between the CNT cantilever and Si substrate, *V*_CNT_, can be assumed to be *V*_*CNT*_ = *V*_0_+*V*_1_*e*^*iωt*^, where *V*_0_ and *V*_1_ respectively stand for the DC and AC components of *V*_CNT_. Assuming that *V*_0_ ~ *V*_s_ and considering only the first order of ω components for simplicity, the AC component *V*_1_, which directly determines the force acting on the CNT cantilever, can be expressed by −*V*_0_*ζη*(*ζ* + *i*)/(1 + *ζ*^2^), where *ζ* = *ωR*_*C*_*C*_0_ and *η* = *C*_1_/*C*_0_(≪1) are the non-dimensional parameters. The imaginary part of this equation causes the retardation. The force acting on the oscillating CNT cantilever is given as 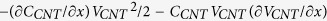 from the charging energy of *C*_CNT_*V*_CNT_^2^/2, where *x* is the position of the CNT cantilever. Under the condition of the harmonically oscillating CNT cantilever with frequency of ω and small amplitude *x*_0_, the position of the CNT cantilever at respective time *t* is definable as 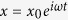. Neglecting the higher order of ω components, the force acting on the CNT cantilever, *F*_*C*_, for the first order oscillation is given approximately as (see Supporting Information for derivation)


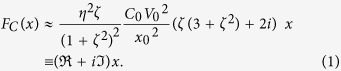


For the simple mass–spring–damper system, the equation of motion with external force *f*(*x*, *t*) is


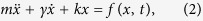


where *m* stands for the mass, γ represents the linear damping factor, and *k* denotes the spring constant. For a harmonically oscillating system with 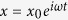, the imaginary part is appeared as *i*ω at the velocity-dependent term corresponding to the Q-factor in eq. 2. Therefore, the imaginary term in eq. 1 is the damping factor in the velocity-dependent term in eq. 2. By substituting eq 1 into eq. 2 as the component of the external force, the equation of motion with forced excitation with *F*_0_*e*^*i*ω*t*^ is


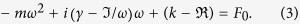


As might be readily apparent in eq. 3, both the linear damping factor and the effective spring constant decrease under the presence of the retardation caused by the pileup charge on the insulator, which improves the Q-factor and lowers the resonance frequency. Consequently, the experimentally obtained result presented in Fig. 1d can be explained qualitatively using the simple mass–spring–damper system with the retardation effect described by the equivalent circuit shown in Fig. 1e.

Figure 2a,b respectively show the electron dose, *q*_ir_, dependence of the frequency response curves of the CNT resonator with the different drive amplitudes of *V*_amp_ = 1 and 3 *V*_pp_. Nonlinear behaviors are clearly observed on the curves. They change depending on the doses and drive amplitude. Nonlinear behavior was hardly observed in the case of the CNT resonator supported by conductive substrate measured by SEM or under the no-charging effect measured using optical method. Consequently, the pileup charge induced by the electron beam should be the origin of the nonlinear behaviors. The complex resonance frequency shifts caused by the pileup charge might be induced by the combination of the retardation effect explained in eq. 3 and the modification of effective spring constant induced by the nonuniformity of the electrostatic potential induced by the pileup charge. Additionally, the duffing type frequency response curve was observed in our experiment. In this case, the frequency at the maximum amplitude is strongly affected by the nonlinearity, which should be considered to be one of origins of the resonance frequency fluctuation. Additional study is necessary for detailed and quantitative understanding of the effect of the pileup charge on the resonance.

Nonlinear phenomena are well known to show large amplitude when the effective spring constant *k*_*eff*_ is expressed as *k*_*eff*_ = *k* + *βx*^2^, where *β* is the nonlinear parameter. The peak shifts toward a higher or lower frequency for *β*  > 0 and *β*  < 0, respectively. The respective nonlinearities are so-called “hardening” and “softening” nonlinearities. Although *β* is usually a material dependent parameter, the external potential field also perturbs the effective spring constant, which results in the modification of both of *k* and *β*. At *q*_ir_ < 6 nC, hardening nonlinearity is observed at both of *V*_amp_ = 1 and 3 *V*_pp_. At *q*_ir_ > 6 nC and *V*_amp_ = 1 *V*_pp_, the nonlinearity vanishes and becomes a linear response. For *V*_amp_ = 3 *V*_pp_, although the response becomes linear at *q*_ir_ ~ 6.2 nC, the softening nonlinear response appears at higher *q*_ir_. Therefore, we conclude that the pileup charge modifies the nonlinear parameter, which is very similar to the case for electrically controlled microscale resonators^25^,^26^.

Figure 2c portrays the electron dose dependence of the oscillation amplitude. For a small amplitude condition of *V*_amp_ = 1 *V*_pp_, both the oscillation amplitude and Q-factor increase concomitantly with increasing *q*_ir_ at *q*_ir_ < 7 nC. The Q-factor is maximum (~3200) at *q*_ir_ of ~7 nC. A further increase of *q*_ir_ decreases the oscillation amplitude and the Q-factor. The application of further electron dose (*q*_ir_ > 9 nC) causes no vibration state. These results imply that the high-dose electron causes the strong electrostatic attraction and that it restricts the oscillation. For the larger amplitude condition of *V*_amp_ = 3 *V*_pp_, the oscillation amplitude increases concomitantly with increasing *q*_ir_ at *q*_ir_ < 8.4 nC. Further electron dose application halts the oscillation. These closely resemble the case at *V*_amp_ = 1 *V*_pp_; they might result from the strong electrostatic attraction. The apparent Q-factor at *q*_ir_ ~ 8.4 nC is ~3900, even in the softening nonlinear regime.

A possible mechanism for the nonlinearity is the nonlinear part of the retardation, which, for simplicity, was neglected in the discussion presented above. Another possible mechanism is the nonuniform electric field around the CNT cantilever induced by the pileup charge as commonly discussed in electrostatically controlled microscale resonator^25^,^26^. To clarify the pileup charge effects on nonlinear behavior, we compute the electric potential distribution induced by the pileup charge using two-dimensional steady state finite element method (FEM) calculation, where the ratio of the pileup charge on the side and top, *q*_side_/*q*_top_, of SU-8 was changed. This is true because the incident electron beam comes from the top surface of the SU-8, so that the amount of pileup charge at the top surface is expected to be different from that at the side. The potential distribution of *q*_side_/*q*_top_ = 0.01 is shown in Fig. 3a, where the potential at the substrate is ground and the CNT is set to be conductive. From the potential profile, the force acting on the CNT was also calculated as the electrostatic force induced by the nonuniform electric field.

To estimate the force gradient acting on the CNT cantilever, the z-position (height) dependence of the electrostatic force acting on the CNT cantilever was obtained as shown in Fig. 3b with different *q*_side_/*q*_top_. The force gradient is correlated directly to the effective spring constant *k*_eff_. The distance dependence of the force acting on the CNT shows nonlinear dependence with a certain curvature corresponding to the nonlinearity. Figure 3c shows the *q*_side_/*q*_top_ dependence of the second-order (*z*^2^) nonlinear parameter of the effective spring constant, which corresponds to *β* described above. The obtained nonlinear parameter is positive at *q*_side_/*q*_top_ < 1 corresponding to the hardening nonlinearity, although the negative nonlinearity is apparent at *q*_side_/*q*_top_ > 1, corresponding to the softening nonlinearity. Therefore, the change of nonlinearity observed in Fig. 2 originates from the potential distribution change induced by the nonuniform charging effect determined by *q*_side_/*q*_top_. This implies that these Q enhancement and nonlinear effect would be modified or reduced due to the modification of time constant for retardation, if the charge leakage on the SU-8 layer was present, which might be caused by insufficient baking of SU-8. Further study must be needed for accurate understanding and quantitative agreement of this phenomena, such as controlled electric field induced by electrodes near the CNT cantilever.

We have demonstrated the improvement of the apparent Q-factor and the control of nonlinearity of the CNT mechanical resonator using the electrostatic interaction induced by pileup charge in SEM. The apparent Q-factor of the CNT mechanical resonator supported by insulator was drastically improved from approx. 630 to 3200, which originated from retardation of the electrostatic force induced by the contact resistance between the CNT and insulator support and the capacitance formed by the CNT cantilever. The FEM calculation revealed that the change of the nonuniform pileup charge strongly influences the nonlinearity of the resonance of the CNT resonator. The improvement of the apparent Q-factor and the change of the nonlinearity induced by the electrostatic attraction are expected to contribute to the improvement of sensitivity of nanomechanical-resonator based sensors.

## Additional Information

**How to cite this article**: Yasuda, M. *et al.* Oscillation control of carbon nanotube mechanical resonator by electrostatic interaction induced retardation. *Sci. Rep.* **6**, 22600; doi: 10.1038/srep22600 (2016).

## Supplementary Material

Supplementary Information

## Figures and Tables

**Figure 1:**
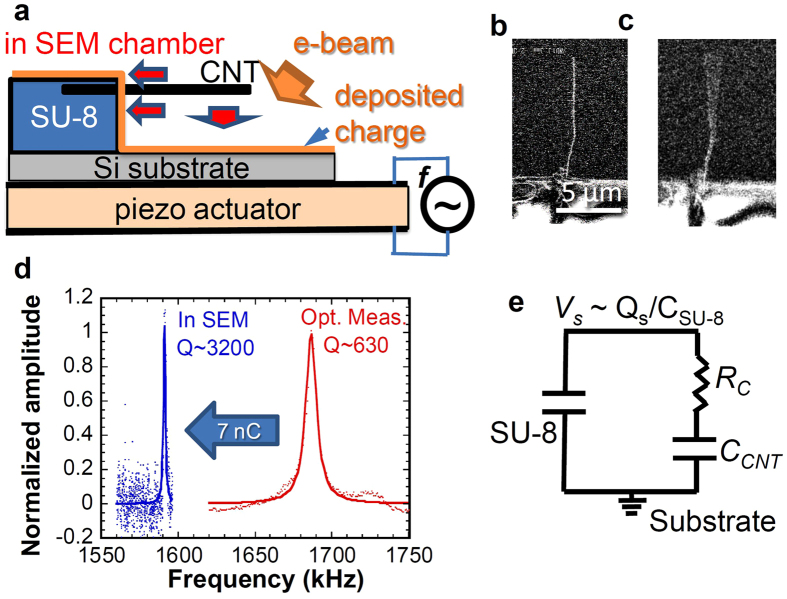
Enhancement of Q-factor of CNT mechanical resonator supported by insulator in SEM. (**a**) Schematic illustration of the experimental setup for the CNT mechanical resonator with insulator support, where thick red arrows indicate the direction of the electrostatic force induced by the deposited charge on insulator indicated by the orange line. SEM images of the CNT mechanical resonator at (**b**) non-resonance and (**c**) resonance. (**d**) Frequency response curves obtained from optical method (red line) and a series of SEM images (blue line) with the electron dose of 7 nC. (**e**) Equivalent circuit for the experimental setup.

**Figure 2:**
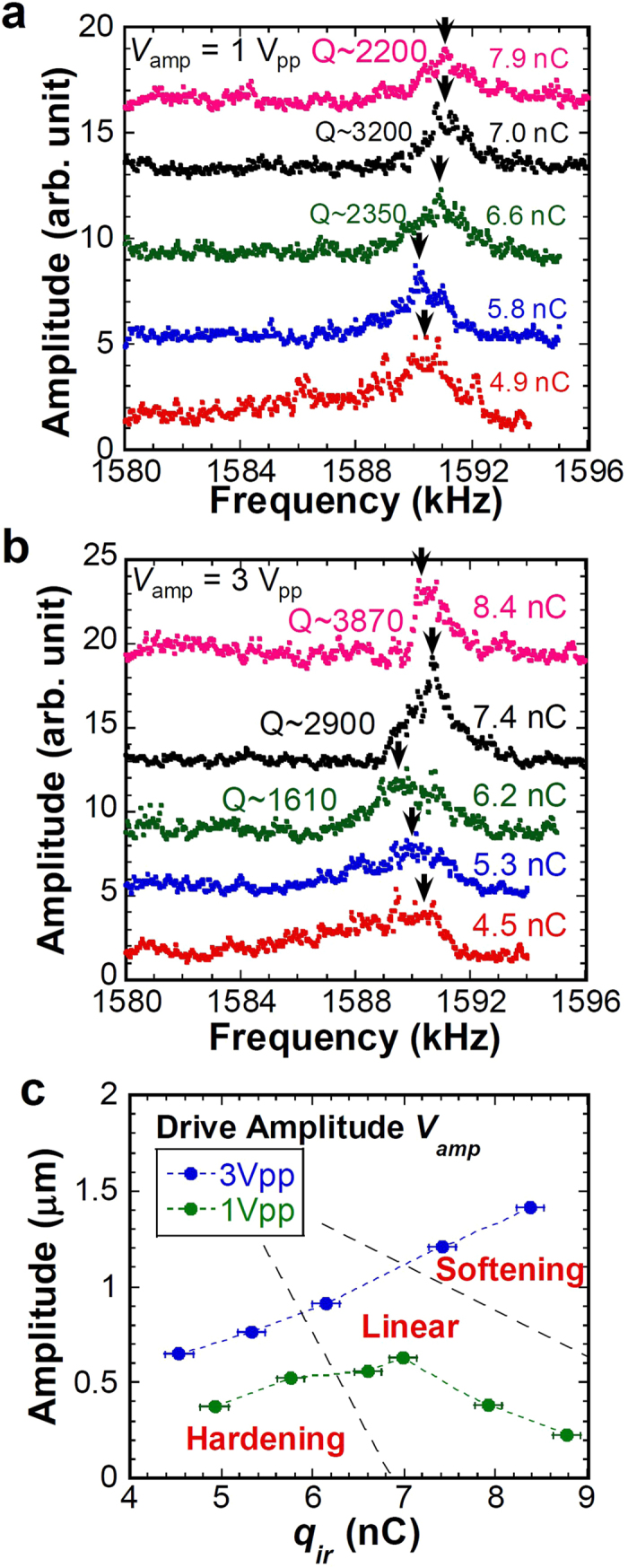
Electron dose dependence of frequency response with different drive amplitudes. (**a,b**) Frequency response curves for *V*_amp_ = 1 and 3 *V*_pp_ under different electron dose conditions. Thick arrows for each curve indicate the resonance frequencies. (**c**) Electron dose dependences of the oscillation amplitude measured from SEM image with *V*_amp_ = 1 and 3 *V*_pp_. Dotted lines are a guide for eyes.

**Figure 3:**
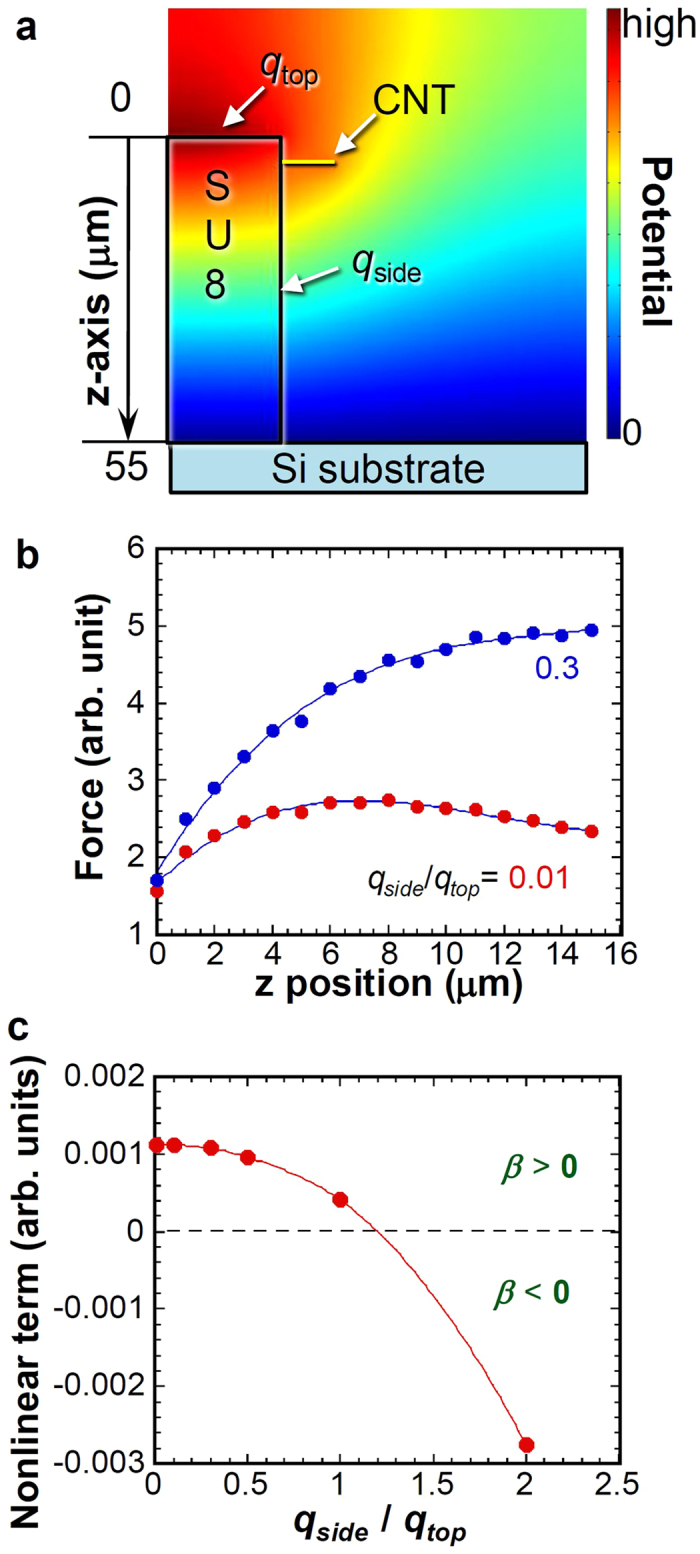
FEM calculations for nonlinear response. (**a**) Setup for FEM calculation and one example of the potential distributions at *q*_side_/*q*_top_ = 0.01. (**b**) Position dependence of the force acting on the CNT cantilever with *q*_side_/*q*_top_ = 0.01 and 0.3. (**c**) Nonuniform charging effect (*q*_side_/*q*_top_) dependence of the nonlinear term of the effective spring constant obtained from position dependence of the force acting on the CNT cantilever by curve fitting.
