# Supplementary material for: Oscillation control of carbon nanotube mechanical resonator by electrostatic interaction induced retardation
Source: Sci Rep. 2016 Mar 3;6:22600. doi: 10.1038/srep22600 (PMC4776282; doi:10.1038/srep22600)
Supplement: Supplementary Information [file srep22600-s1.pdf]

Supplementary Information

Oscillation control of carbon nanotube mechanical resonator by electrostatic interaction induced retardation

Masaaki Yasuda, Kuniharu Takei, Takayuki Arie, and Seiji Akita*

Department of Physics and Electronics, Osaka Prefecture University

1-1 Gakuen-cho, Naka-ku, Sakai 599-8531, Japan

1. Sample fabrication procedure

Figure S1 shows a schematic fabrication procedure for the sample examined here and the detailed procedures are as follows.

1) We prepared dispersed CNTs on a membrane filter through the filtration process of CNT suspension.

2) A first SU-8 layer (~50 μm thick) was formed on a SiO_2/Si substrate by spin coating.

3) The dispersed CNTs on the membrane filter were transferred on the 1st SU-8 layer by stamping the membrane filter, which results in the well-dispersed CNTs on the 1st SU-8 layer.

4) A 2nd SU-8 layer (~5 μm) was formed by spin coating. Thus, the dispersed CNTs were sandwiched by SU-8 layers on a Si substrate.

5) To fabricate the cantilevered CNT supported by SU-8, the root of CNT was exposed by focused laser (~500 nm diameter) with a wavelength of 406 nm, where the exposed part of SU-8 were cross-linked, resulting in insoluble to liquid developers.

* e-mail: akita@pe.osakafu-u.ac.jp

6) After the development for removing the unexposed part, the sample was dried by critical point drying process to prevent the sticking during the drying process and baked at 150 °C. Note that once the SU-8 is exposed to the UV light and baked at 150 °C, the molecules are cross-linked, which results in a very stable form against the electron beam irradiation unlike PMMA.

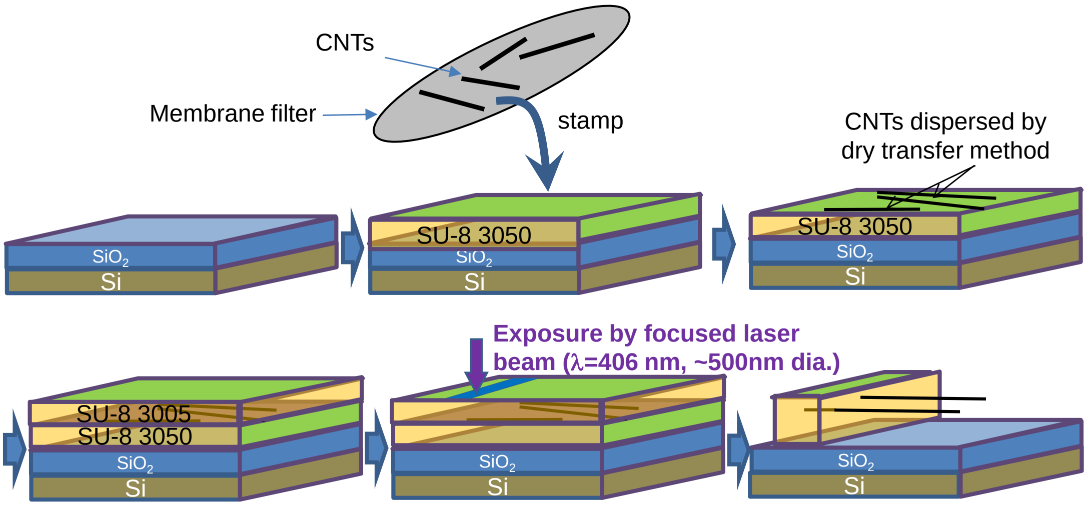

Figure S1. Schematic fabrication procedure of CNT cantilever supported by SU-8.

2. Reproducibility using other CNT mechanical resonator supported by insulator

Figure S2a shows a frequency response curve with and without the pileup charge for the CNT mechanical resonator supported by insulator. The Q-factor obtained from the SEM measurements with the electron dose of approx. 0.8 nC is ~7500. The apparent Q-factor is improved at the presence of the pileup charge. Instead of the piezoelectric actuator, photothermal vibration was used to oscillate the CNT cantilever at the optical measurement. Figure S3b presents an SEM image of the CNT cantilever used in this experiment. The Young's modulus of the CNT estimated from the resonance frequency is ~0.4 TPa, where the diameter and length are, respectively, ~85 nm and 11.5 μm.

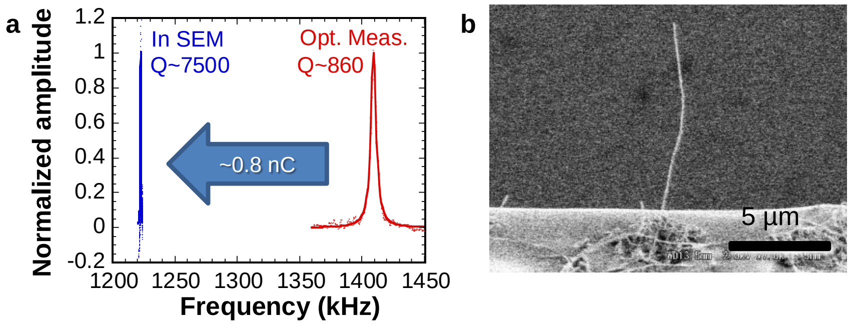

Figure S2. **Another CNT mechanical resonator supported by insulator:** (a) Frequency response curves with and without the pileup charge on the insulator substrate. (b) SEM image of the CNT mechanical resonator.

3. Force acting on CNT cantilever induced by pileup charge on SU-8

As mentioned in main text, the resonance property of CNT resonator supported by a "conductive" substrate (connected to a ground plane) measured in SEM is almost identical to that measured by the optical method. Figure S3 shows one of examples for the comparison, where the support of the CNT cantilever was coated by Au. Note that the observed slight shift of resonance frequency is most likely due to some contaminants in air during the sample transfer. Only in the case of the "insulator" support, we found the improvement of Q factor and the nonlinear response.

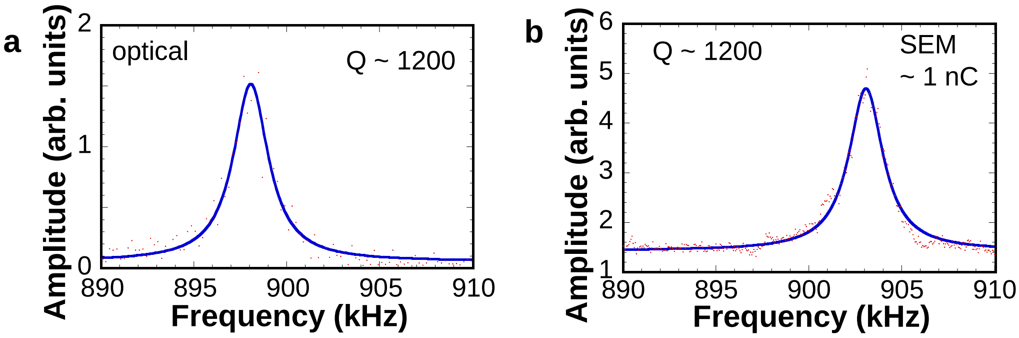

Figure S3. Resonance properties of CNT cantilever supported by conductive substrate: Resonance curves measured by (a) optical method and (b) SEM images.

The bending CNT shown in Fig. S4a was used for the estimation of force acting on the CNT cantilever induced by the pileup charge in this experiment. In order to obtain the force acting on the CNT cantilever, the deflection of the CNT cantilever tip was measured after applying the additional electron dose of ~0.7 nC. The additional electron dose caused ~86 nm deflection of the CNT cantilever tip, where the deflection was obtained from the curve fitting (Gaussian distribution) of brightness distribution of the SEM images at the tip of the CNT as shown in Fig. S4b. As a result, the force acting on the CNT cantilever was estimated to be 0.3 nN from the deflection, Young's modulus of the CNT (0.18 TPa) measured from resonance frequency, and the size (9 μm in length and 100 nm in diameter) of the CNT. Note that the quantitative comparison to other CNTs is still difficult because of the strong surface condition dependence of the pileup charge.

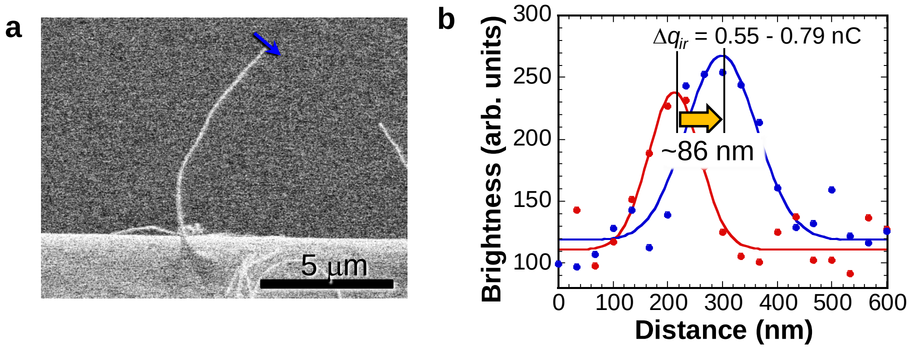

Figure S4. Force acting on CNT cantilever induced by pileup charge on SU-8: (a) SEM image of CNT cantilever supported by SU-8. A blue arrow indicates the deflection direction of the CNT cantilever tip after the application of additional electron dose. (b) Brightness distributions along the blue arrow of the SEM image around the tip of the CNT cantilever before (red) and after (blue) the application of the additional electron dose, where the solid lines are fitted curve to the experimental data to obtain the tip position.

4. Defect effect on CNT

CVD grown CNTs usually contain many defects including kink structure. In this case, however, not only nonlinear behavior but also Q-enhancement were hardly observed in absence of pileup charge even with the kink-CNT cantilever. From the point of view of the multiple defects, individual CNTs should be treated as a series connection of n-th springs with different spring constant, k_1 .... k_n. In the case of series connection of springs, the effective spring constants k_{eff} is given by $k_{eff}^{-1} = k_1^{-1} +$ .... k_n^{-1}.[1] In the case of CNTs with kink defects, the defect part at the kink can also be treated as the small spring which is serially connected to other part of CNT. These coupled spring system can be considered as one-spring with linear response under small vibration amplitude regime as mentioned above, where the coupled effective spring constant k_{eff} was different from original system and have different Eigen frequencies. Thus, the defects in the CNT do not induce apparent nonlinear or Q-factor enhancement effect under linear elastic regime.

In order to clarify the effect of kink defect, we performed a finite element method (FEM) calculation for cantilever with kink defect as shown in Fig. S5a, where a crack at kink portion was introduced for defect model. As apparently observed in Fig. S5b, the forces acting on the cantilevers for both cases show linear response with the deflections, while the effective spring constant for cantilever with a crack defect is smaller. Thus, the defects simply induce the modification of the effective spring constant at small deflection regime corresponding to our experimental condition.

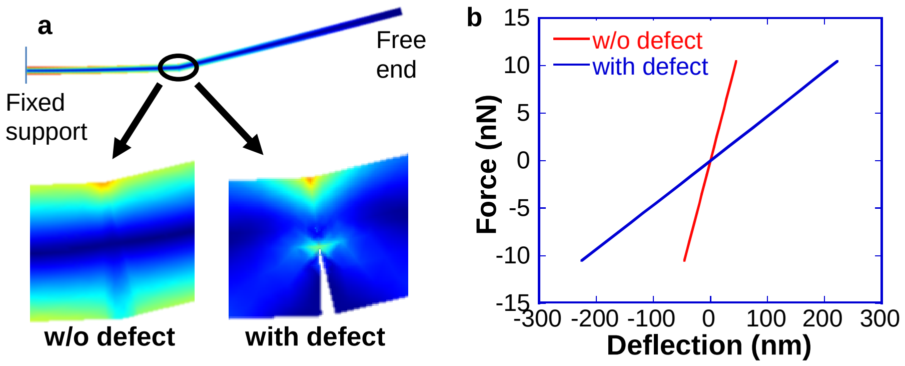

Figure S5. FEM calculations for defect effect: (a) FEM models for with or without a crack defect. (b) Deflection at free end of cantilever dependence of force acting on the cantilever.

5. Derivation of retardation effect on force acting on CNT

Figure 1a in the main text shows that the cantilevered CNT mechanical resonator is supported by a SU-8 photoresist, which is an insulator. The simplified equivalent circuit of this device setup is shown in Figure 1e in the main text, where the capacitance of the SU-8, $C_{\mathrm{SU\text{-}8}}$, is much greater than that of the oscillating CNT resonator, C_{CNT}, which results in almost constant voltage V_{s} at the SU-8 surface, even with CNT cantilever oscillation. The CNT cantilever is connected to the constant voltage source V_{s} through a certain contact resistance R_{C}. Assuming a harmonically oscillating CNT cantilever with the linear response regime, capacitance C_{CNT} modulated during the oscillation with frequency ω at a certain time t for the first order can be expressed as

$$C_{CNT} \approx C_0 + C_1 e^{i\omega t}, \quad \text{(S1)}$$

where C_0 and C_1 respectively stand for the DC and AC components of C_{CNT}. From the equivalent circuit shown in Figure 1e in main text, the total potential induced at R_{C} and C_{CNT} is expected to be equal to V_{s}, expressed as

$$\begin{aligned} V_S &= V_{CNT} + R_C \frac{\partial(C_{CNT}V_{CNT})}{\partial t} \\ &= V_{CNT} + R_C \left[V_{CNT} \frac{\partial C_{CNT}}{\partial t} + C_{CNT} \frac{\partial V_{CNT}}{\partial t} \right] \\ &= V_{CNT} + R_C \left[i\omega V_{CNT} C_1 e^{i\omega t} + \left(C_0 + C_1 e^{i\omega t} \right) \frac{\partial V_{CNT}}{\partial t} \right] \end{aligned} . \quad \text{(S2)}$$

Solving the differential equation for V_{CNT}, the solution is given as

$$V_{CNT}(t) = \frac{A e^{-\frac{t}{C_0 R_C}}}{(C_0 + C_1 e^{i\omega t})^{1+i\frac{1}{\omega C_0 R_C}}} + V_S \, {}_2F_1\left[(1,1; \left(1 - i\frac{1}{\omega C_0 R_C} \right); -\frac{C_1}{C_0} e^{i\omega t} \right], \quad \text{(S3)}$$

where ${}_2F_1$ is the hypergeometric function given by

$${}_2F_1(a,b;c;z) = \sum_{n=0}^{\infty} \frac{(a)_n (b)_n}{(c)_n} \frac{z^n}{n!}, \text{ where } (q)_n = \begin{cases} 1 & n=0 \\ q(q+1)\cdots(q+n-1) & n>0 \end{cases},$$

and A signifies the constant of integration. The first term in right-hand side of eq. S3 is the transient response term. Here, we particularly examine the harmonically oscillating state after the transient state, so that the first term disappears after sufficient time (large t). Under the condition of $C_1/C_0 << 1$ corresponding to the small vibration amplitude, function $V_{\mathrm{CNT}}(t)$ can be approximated as

$$V_{CNT}(t) = V_0 + V_1 e^{i\omega t}, \quad \text{(S4)}$$

where $V_0 \sim V_s$ and V_1 respectively denote the DC and AC components of V_{CNT}. Inserting eq. S4 into eq. S2, eq. 2 becomes

$$\begin{aligned} 0 &= V_1 e^{i\omega t} + R_C \left[i\omega (V_0 + V_1 e^{i\omega t}) C_1 e^{i\omega t} + \left(C_0 + C_1 e^{i\omega t} \right) i\omega V_1 e^{i\omega t} \right] \\ &= \left(V_1 + i\omega C_1 R_C V_0 + i\omega C_0 R_C V_1 \right) e^{i\omega t} + i2\omega V_1 C_1 R_C e^{i2\omega t} \end{aligned} . \quad \text{(S5)}$$

Considering only the first order of ω components for simplicity (this is reasonable because of $C_1 V_1 << 1$), the AC component V_1 can be expressed as

$$V_1 = -V_0 \zeta \eta (\zeta + i)/(1 + \zeta^2), \quad \text{(S6)}$$

where $\zeta = \omega R_C C_0$ and $\eta = C_1 / C_0$ are the non-dimensional parameters. From the charging

energy of $C_{CNT}V_{CNT}^2/2$ between the oscillating CNT cantilever and the substrate, the force acting on the oscillating CNT cantilever $F_C(x)$ is given as

$$F_C(x) = -(\partial C_{CNT}/\partial x)V_{CNT}^{\ 2}/2 - C_{CNT}V_{CNT}(\partial V_{CNT}/\partial x)\,, \tag{S7}$$

where x is the CNT cantilever position. Under the condition of the harmonically oscillating CNT cantilever with a frequency of ω and an small amplitude x_0, the position of the CNT cantilever at respective time t is definable as $x = x_0 e^{i\omega t}$. Using this relation and inserting eqs. S1, S4, and S6 into eq. S7, one obtains

$$F_C(x) = \frac{C_1(-ix_0 + x_0\zeta - x\zeta\eta C_0)(ix_0 + x_0\zeta + 3x\zeta\eta)V_0^{\ 2}}{2x_0^{\ 3}(-i+\zeta)}\,. \tag{S8}$$

$x = x_0 e^{i\omega t}$Neglecting the terms which are independent from position x and higher order of x^2 components, the force acting on the CNT cantilever, F_C, for the first order, which is proportional to x, is given as

$$\begin{aligned} F_C(x) &\approx \frac{\eta^2\zeta}{(1+\zeta^2)^2}\frac{C_0V_0^{\ 2}}{x_0^{\ 2}}\left(\zeta(3+\zeta^2)+2i\right)x \\ &\equiv (\Re + i\Im)x \end{aligned}\,. \tag{S9}$$

This equation is the same as eq. 1 in the main text.

Reference

1 Lee, K. B. *Principles of Microelectromechanical Systems*. 271-310 (Jhon Wiley & Sons, Inc., 2011).
